# Shifts in temperature within the physiologic range modify strand-specific expression of select human microRNAs

**DOI:** 10.1261/rna.049122.114

**Published:** 2015-07

**Authors:** Ratnakar Potla, Ishwar S. Singh, Sergei P. Atamas, Jeffrey D. Hasday

**Affiliations:** 1 Pulmonary and Critical Care Medicine Division, University of Maryland School of Medicine, Baltimore, Maryland 21201, USA; 2 Medicine and Research Services, Baltimore VA Medical Center, Baltimore, Maryland 21201, USA; 3 Division of Rheumatology and Clinical Immunology, Department of Medicine, University of Maryland School of Medicine, Baltimore, Maryland 21201, USA

**Keywords:** microRNA, hyperthermia, hypothermia, lung epithelium

## Abstract

Previous studies have revealed that clinically relevant changes in temperature modify clinically relevant gene expression profiles through transcriptional regulation. Temperature dependence of post-transcriptional regulation, specifically, through expression of miRNAs has been less studied. We comprehensively analyzed the effect of 24 h exposure to 32°C or 39.5°C on miRNA expression profile in primary cultured human small airway epithelial cells (hSAECs) and its impact on expression of a targeted protein, protein kinase C α (PKCα). Using microarray, and solution hybridization-based nCounter assays, with confirmation by quantitative RT-PCR, we found significant temperature-dependent changes in expression level of only five mature human miRNAs, representing only 1% of detected miRNAs. Four of these five miRNAs are the less abundant passenger (star) strands. They exhibited a similar pattern of increased expression at 32°C and reduced expression at 39.5°C relative to 37°C. As PKCα mRNA has multiple potential binding sites for three of these miRNAs, we analyzed PKCα protein expression in HEK 293T cells and hSAECs. PKCα protein levels were lowest at 32°C and highest at 39.5°C and specific miRNA inhibitors reduced these effects. Finally, we analyzed cell-cycle progression in hSAECs and found 32°C cells exhibited the greatest G1 to S transition, a process known to be inhibited by PKCα, and the effect was mitigated by specific miRNA inhibitors. These results demonstrate that exposure to clinically relevant hypothermia or hyperthermia modifies expression of a narrow subset of miRNAs and impacts expression of at least one signaling protein involved in multiple important cellular processes.

## INTRODUCTION

Human core body temperature is homeostatically maintained within a narrow range (Morrison and Nakamura 2011). The surface-to-core temperature gradient increases during cold exposure, extending to subcutaneous and muscle tissues up to 4 cm deep (Webb 1992). Core temperature itself can increase by several degrees during fever (Singh and Hasday 2013), exertional/environmental hyperthermia (Leon and Helwig 2010), or as part of adverse drug reactions (Hopkins 2011). A decrease by several degrees may occur during accidental (Reed 1996) or therapeutic hypothermia (The Hypothermia after Cardiac Arrest Study Group 2002; Bernard et al. 2002), sepsis (Clemmer et al. 1992), trauma (Jurkovich et al. 1987), or certain drug intoxications (van Marum et al. 2007; Wilson and Waring 2007). Solid organs harvested for transplantation may be stored at more extreme hypothermia for prolonged periods prior to transplant. Work from our group and others has shown that modest changes in temperature within the physiological range alters expression level of certain genes with important physiologic and clinical consequences (Cahill et al. 1996; 1997; Chen et al. 1997, 2006, 2009; Wang et al. 1998; Jiang et al. 1999a,b, 2000; Fairchild et al. 2000; Singh et al. 2000, 2002, 2008; Hasday et al. 2003; Ellis et al. 2005; Appenheimer et al. 2007; Vardam et al. 2007; McClung et al. 2008; Nagarsekar et al. 2008, 2012; Cooper et al. 2010a,b; Lipke et al. 2010, 2011; Fisher et al. 2011; Maity et al. 2011; Tulapurkar et al. 2011, 2012; Shah et al. 2012; Zhang et al. 2012; Gupta et al. 2013). Most of the studies of temperature-dependent gene expression have focused on transcriptional regulation (Cahill et al. 1996, 1997; Chen et al. 1997; Singh et al. 2008; Cooper et al. 2010a,b; Maity et al. 2011; Zhang et al. 2012), but post-transcriptional regulation by small noncoding RNAs like microRNA (miRNA) may be as important as transcriptional regulation for ∼30% of all genes (Lewis et al. 2005).

The classical pathway to generation of mature miRNAs is a stepwise process that progresses from primary transcripts (pri-miRNAs) that fold into imperfect dsRNA-like hairpins by sequential action of two RNase III family nucleases. In the first step, a Class 2 RNase III enzyme Drosha cleaves the sequences 5′ and 3′ to the hairpin structure releasing the ∼70 nt pre-miRNA, which contains the mature miRNA duplex within its hairpin structure. The pre-miRNAs are exported to the cytoplasm and incorporated into the RNA-induced Silencing Complex (RISC) where the endonuclease Dicer cleaves the loop structures to yield ∼21 nt miRNA duplexes with protruding 2 nt 3′ ends (for review, see Filipowicz et al. 2008). Only one strand of the duplex is retained as the mature miRNA in the RISC and the other is usually degraded (Schwarz et al. 2003). Considering this study is focused on temperature-dependent miRNA expression, it is noteworthy that the strand with the 5′ terminus located at the thermodynamically less-stable end of the duplex is usually retained in the RISC as the mature miRNA (Khvorova et al. 2003).

Based on previous studies showing that transcription of certain mRNAs is temperature-dependent and considering the reported importance of thermodynamics to miRNA strand selection, we hypothesized that changes in temperature within physiological range may also alter expression of certain mature miRNAs, which might have impact on expression of gene products with important potential consequences for homeostasis and disease pathogenesis. We utilized two complementary methods to analyze miRNA expression profiles in primary cultured human small airway epithelial cells (hSAECs) and Human Embryonic Kidney cells (HEK 293T) incubated at 32°C, 37°C, and 39.5°C. We confirmed temperature-responsive miRNA expression using quantitative RT-PCR, performed an in silico analysis of potential targets of multiple temperature-responsive miRNAs, and analyzed the impact of temperature changes on protein expression of PKCα, an important signaling molecule and predicted target of multiple temperature-sensitive miRNAs.

## RESULTS

### Mature human miRNA detection by both platforms

Human SAECs were incubated for 24 h with or without 1 ng/mL TNFα at 32°C, 37°C, or 39.5°C and total RNA was isolated. RNA samples were analyzed for quality by capillary electrophoresis. Samples from four experiments were found to be high quality and were analyzed for miRNA expression profile. RNA samples from all four experiments were analyzed for miRNA profile using the nCounter miRNA assay. Samples from three of these experiments were also analyzed by Affymetrix miRNA microarray. The microarray and nCounter platforms comprised 632 and 811 probe sets, respectively, for mature human miRNAs. The two platforms shared 425 common probe sets. Of the 386 unique miRNAs probed by the nCounter miRNA assay, 64 were detected in at least one sample in all four sample sets. Of the 207 unique miRNAs probed by microarray, 131 were detected in at least one sample in all three sample sets. Of the 425 common miRNAs, 139 were detected by both platforms, 18 of which were detected only by nCounter, and 153 only by microarray. Thus of the 1018 miRNAs probed, 505 (49.6%) were detected in at least one sample per experiment by one or both assays.

### Identification of differentially expressed miRNA

**FIGURE 1.**
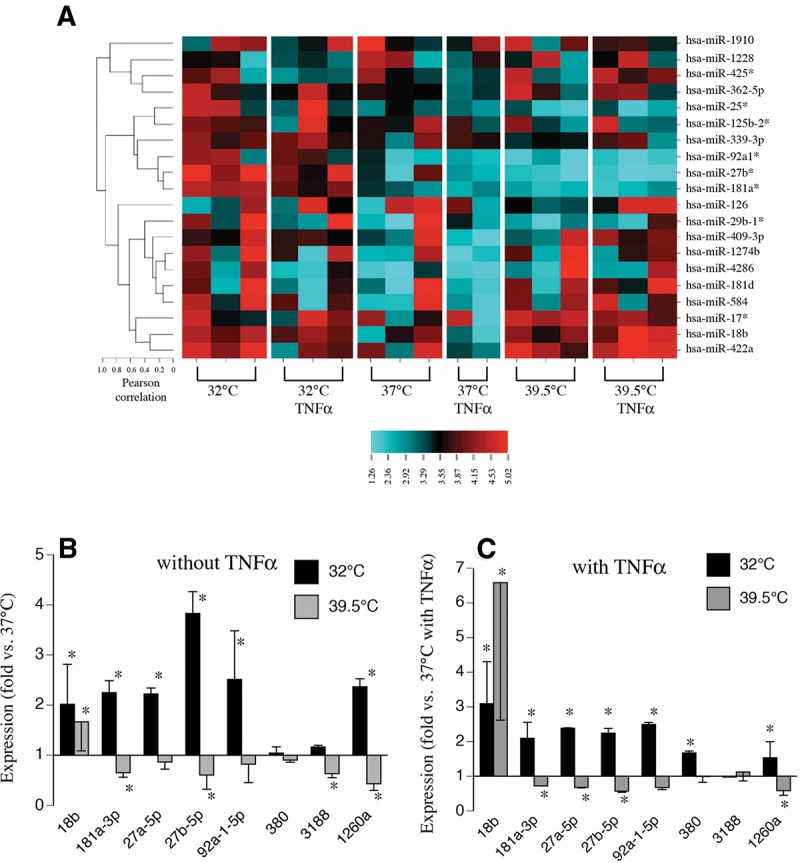
Microarray and nCounter analysis of the effects of incubation temperature on miRNA expression pattern in human SAECs. (*A*) HEATMAP of selected miRNAs detected by microarray in hSAECs following 24 h incubation at 32°C, 37°C, or 39.5°C in the absence or presence of 1 ng/mL rhTNFα. (*B*,*C*) Microarray data for seven miRNAs exhibiting temperature-dependent expression and nCounter data for miR1260a expression expressed as fold-change versus 37°C cells in the absence (*B*) or presence (*C*) of TNFα. Mean ± SEM. (*) *P* < 0.01 versus 37°C. *N* = 3 for microarray and 4 for nCounter.

To model accidental or therapeutic hypothermia or exertional/environmental hyperthermia, hSAECs were exposed to 32°C or 39.5°C, respectively, in the absence of TNFα. To model sepsis-related hypothermia or fever these cells were exposed to 32°C or 39.5°C, respectively, in the presence of 1 ng/mL TNFα, a proinflammatory cytokine that plays a central role in the pathogenesis of sepsis (Qiu et al. 2011). Of the 505 miRNAs detected in hSAECs, only 11 were differentially expressed in cells incubated for 24 h at 32°C and/or 39.5°C with or without TNFα (Table 1; Fig. 1A). Of these, 10 were detected by microarray and one was detected by nCounter. Seven of the temperature-responsive miRNAs that were detected by microarray were not included in the nCounter probe set. The other three miRNAs found to be temperature-responsive by microarray, hsa-miR-3195, hsa-miR-1468, and hsa-miR-564, were present in the nCounter probe set but were not detected in any of the samples by nCounter assay and were not included in the subsequent analysis. For hsa-miR-18b, the temperature-response pattern was different in the presence or absence of TNFα. Without TNFα, hsa-miR-18b expression levels were reduced at both 32°C and 39.5°C compared with 37°C, but when the hSAECs were treated with TNFα, miR-18b expression levels were higher at both 32°C and 39.5°C compared with 37°C. For the other six temperature-responsive miRNAs identified by microarray, the temperature-response pattern was unaffected by the presence or absence of TNFα. Four of these miRNAs, hsa-miR-181a-3p, hsa-miR-27a-5p, hsa-miR-27b-5p, and hsa-miR-92a-1-5p, exhibited a similar expression pattern with highest expression at 32°C and lowest at 39.5°C. For the other two miRNAs, hsa-miR-380-5p and hsa-miR-3188, gene expression was lower at both 32°C and 39.5°C than at 37°C (Fig. 1B,C). The only temperature-responsive miRNA detected exclusively by the nCounter miRNA assay, miR-1260a, exhibited a temperature-response pattern similar to miR-181a, and two-way ANOVA analysis showed there was no effect of TNFα.

**TABLE 1.**
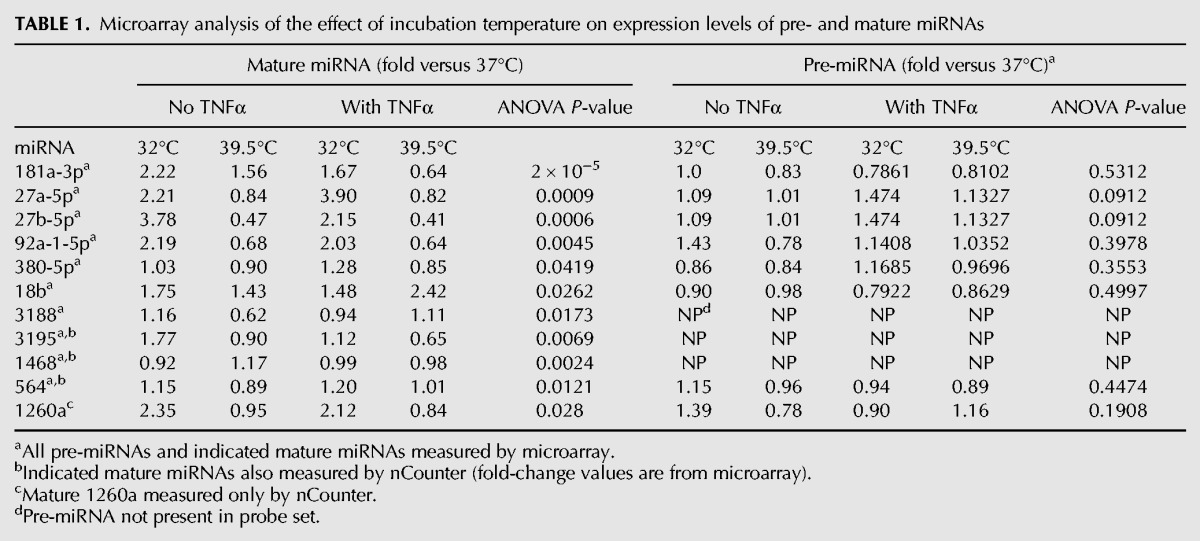
Microarray analysis of the effect of incubation temperature on expression levels of pre- and mature miRNAs

### Confirmation of selected miRNA by quantitative PCR

**FIGURE 2.**
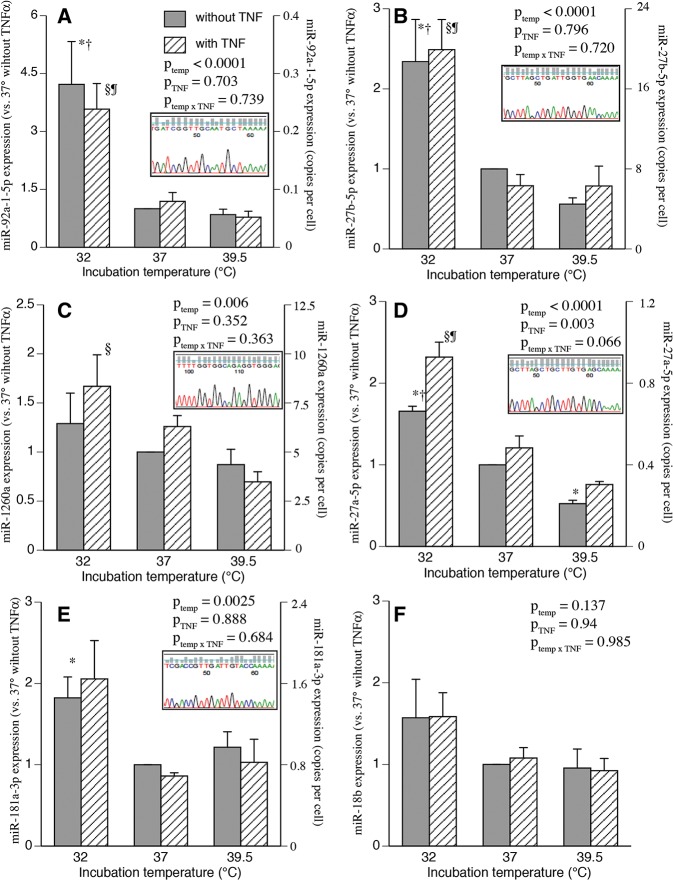
Quantitative RT-PCR confirmation of temperature effects on miRNA expression pattern in human SAECs. Human SAECs from two lots were incubated for 24 h at 32°C, 37°C, or 39.5°C without or with 1 ng/mL rhTNFα. Total RNA was collected and levels (*A*) hsa-miR-92a-1-5p, (*B*) hsa-miR-27b-5p, (*C*) hsa-miR-1260a, (*D*) hsa-miR-27a-5p, (*E*) hsa-miR-181a-3p, and (*F*) hsa-miR-18b were analyzed by qRT-PCR as described in Materials and Methods and expressed as a fold-change versus 37°C cells without TNFα. The *right*-hand axis displays the total expression level in copies per cell, which was calculated as described in Materials and Methods. Mean ± SEM. (*) *P* < 0.05 versus 37°C; (†) *P* < 0.05 versus 39.5°C; (§) *P* < 0.05 versus 37°C with TNFα; (¶) *P* < 0.05 versus 39.5°C with TNFα, *n* = 4. The temperature-responsive miRNAs were cloned into T-Vector and sequenced (*inset*).

The six miRNAs (hsa-miR-18b, hsa-miR-27a-5p, hsa-miR-27b-5p, hsa-miR-92a-1-5p, hsa-miR-181a-3p, and hsa-miR-1260a) shown to exhibit consistent patterns of temperature-dependent expression by microarray and nCounter assays were further analyzed by qRT-PCR. Five of these miRNAs (hsa-miR-27b-5p, hsa-miR-92a-1-5p, hsa-miR-27a-5p, hsa-miR-181a-3p, and hsa-miR-1260a) showed similar patterns of temperature-responsiveness by qRT-PCR and microarray or NCounter analysis (Fig. 2A–E). The identities of each qRT-PCR product were confirmed to be the miRNA of interest by cloning and sequencing; the chromatograms for each are shown in Figure 2. The absolute expression level of each of the five temperature-responsive miRNAs was estimated from the Nanostring data and is shown on the right-hand *y*-axis in the Figure 2A–E. The temperature-responsiveness demonstrated for hsa-miR-18b by microarray was not confirmed by qRT-PCR (Fig. 2F). Four of these confirmed miRNAs represent the star strand, which is accepted as the less abundant strand for the corresponding mature miRNA duplex (Schwarz et al. 2003).

**FIGURE 3.**
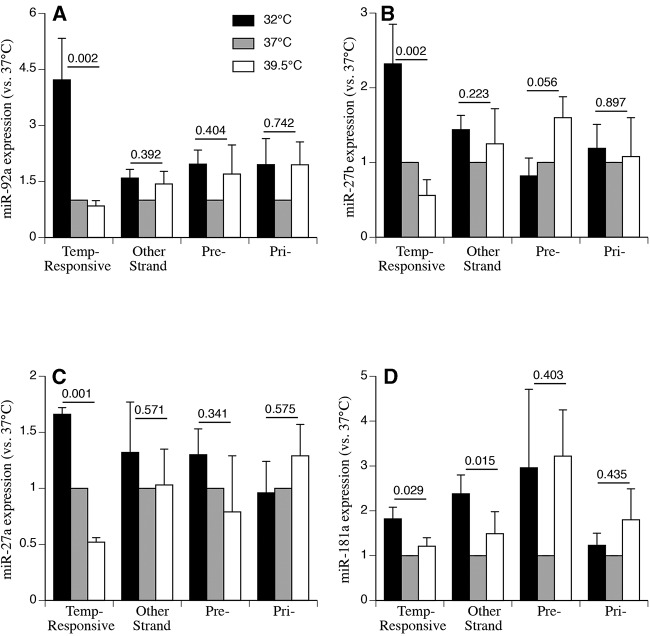
Quantitative RT-PCR analysis of temperature effects on pri-miRNA, pre-miRNA, and opposite strand expression pattern in human SAECs. Human SAECs from two lots were incubated for 24 h at 32°C, 37°C, or 39.5°C without rhTNFα. Total RNA was collected and levels of the temperature-responsive strand, the sister strand, the pri-miRNA, and the pre-miRNA for (*A*) hsa-miR-92a-1, (*B*) hsa-miR-27b, (*C*) hsa-miR-27a, and (*D*) hsa-miR-181a were analyzed by qRT-PCR as described in Materials and Methods and are expressed relative to expression of each in 37°C hSEAECs. Mean ± SEM, *n* = 4. The *P*-values from one-way ANOVA are indicated.

Levels of the opposite miRNA strand and corresponding long form stem-loop precursors (pre-miRNA), and primary transcripts (pri-miRNA) for four of the five confirmed temperature-responsive miRNAs were quantified by qRT-PCR (Fig. 3). A similar analysis of hsa-miR-1260a was not possible as its pre-miRNA sequence is embedded in exon 3 of the neuroglobin gene and its 3p arm has not yet been defined. The corresponding opposite strands, the pre-, and pri-forms for three of these miRNAs, hsa-miR-92a-3p, hsa-miR-27b-3p, and hsa-miR-27a-3p, were not affected by either temperature change (Fig. 3A–C) or TNFα treatment (data not shown). In contrast, for hsa-miR-181a-5p, expression levels of both mature strands, pre- and pri-forms tended to be higher at both 32°C and 39.5°C compared with 37°C (Fig. 3D). In addition, pre-miRNA forms for three of the temperature-sensitive miRNAs, hsa-miR-92a, hsa-miR-27a, and hsa-miR-1260a, were included in the microarray probe set and shown to have similar expression levels at all three temperatures (Table 1).

### Thermodynamic properties of temperature-responsive miRNAs

We analyzed the mature miRNA duplexes corresponding to four of the five temperature-responsive miRNAs for predicted overall duplex stability, the stability of each end of the duplex, the asymmetry of stability between ends of each duplex, and its relationship to the mature temperature-responsive miRNA strand. We compared each of the temperature-responsive miRNAs with the other non-temperature-responsive miRNAs from the same cluster, which were identified using mIRBase (Table 2; Kozomara and Griffiths-Jones 2014). A similar analysis of hsa-miR-1260a was not possible because its p-arm has not been unambiguously identified. Hsa-miR-27a and hsa-miR-27b were the most stable miRNAs in their respective clusters and hsa-miR-92a-1 was the second most stable of six miRNAs in its cluster. There was only one other miRNA in the hsa-miR-181a-1 cluster, hsa-miR-181b-1, and both miRNAs had the same predicted free energy. Three of the four temperature-responsive miRNAs exhibited the greatest asymmetry in predicted stability between duplex ends within their cluster. Interestingly, all four of the temperature-responsive miRNAs were derived from the strand with greater duplex stability at its 5′ end and, therefore, predicted to be the less abundant or star strand (Khvorova et al. 2003).

**TABLE 2.**
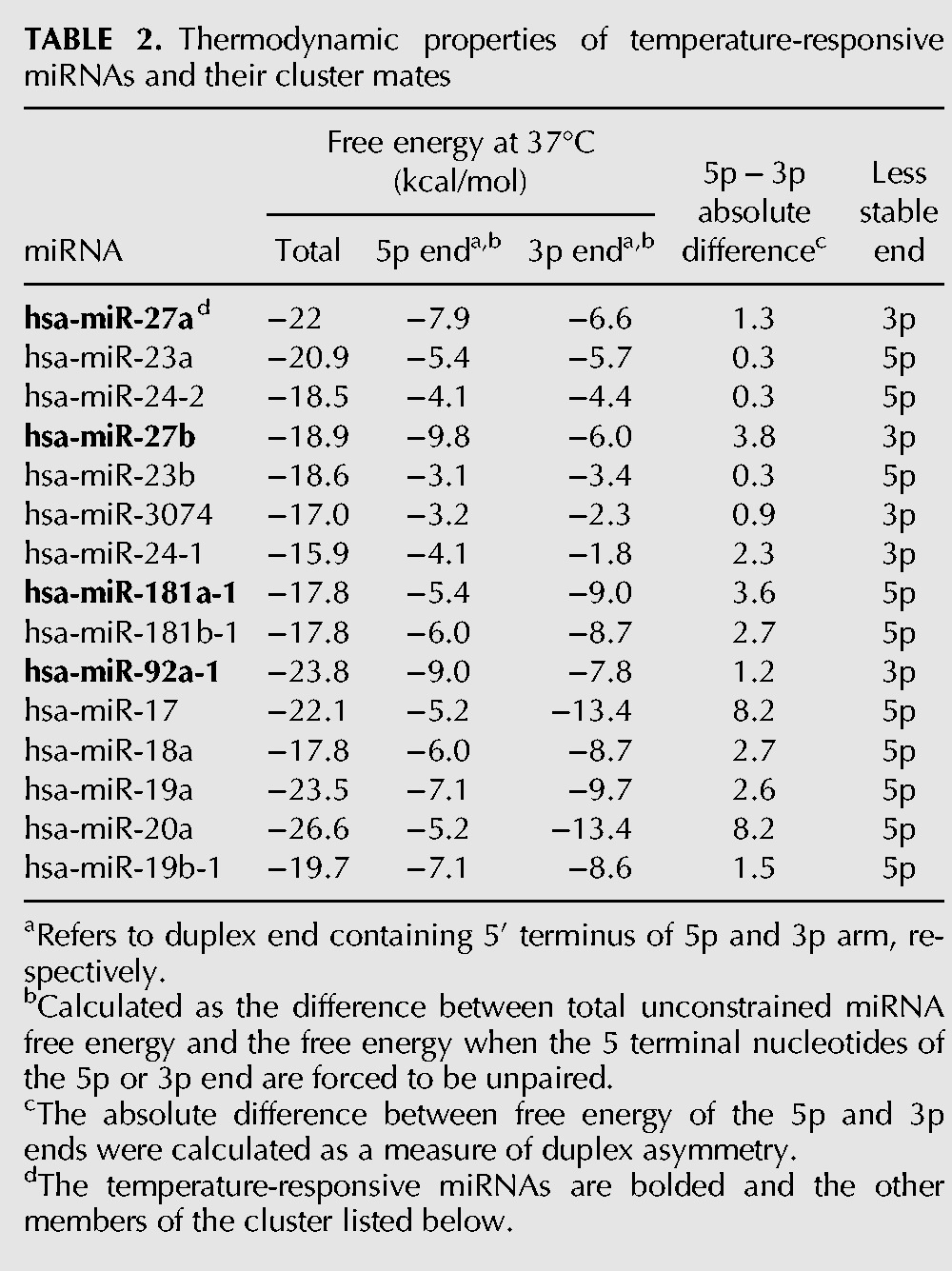
Thermodynamic properties of temperature-responsive miRNAs and their cluster mates

### Target prediction and validation

**FIGURE 4.**
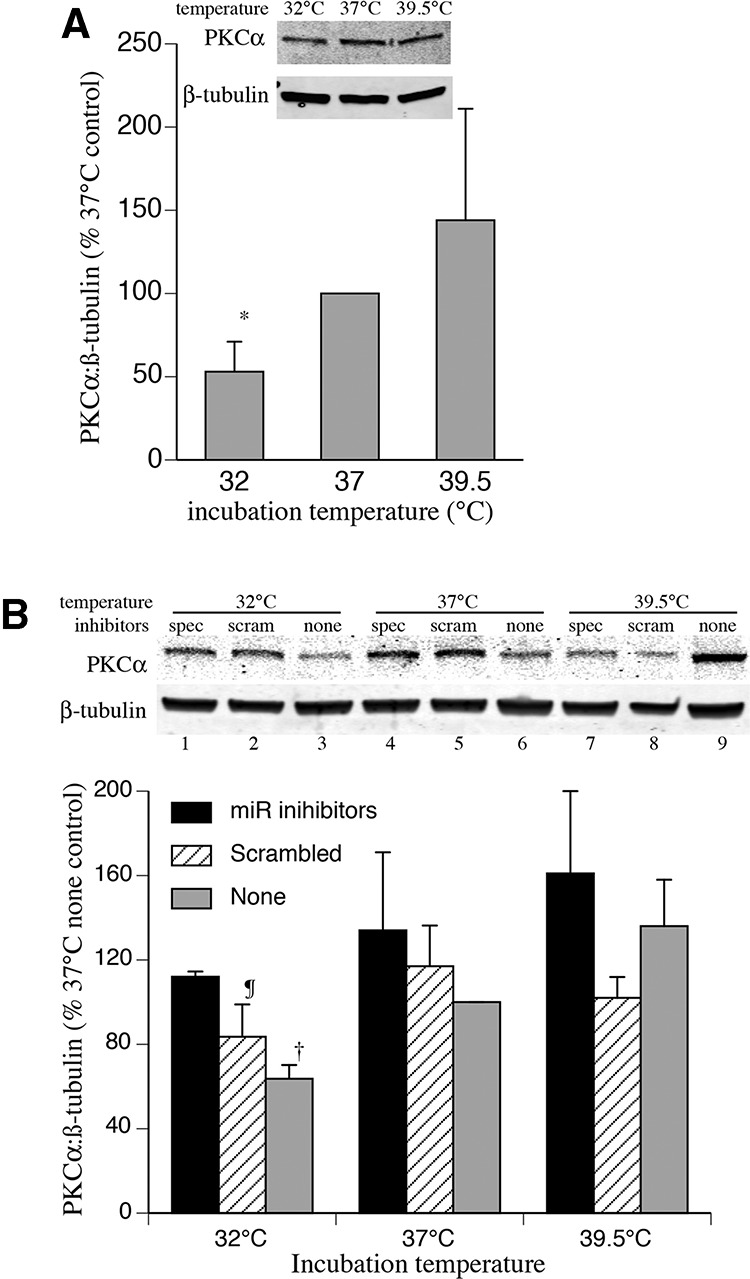
Clinically relevant temperature change-induced expression of miRNAs alters the PKCα protein levels. (*A*) Human SAECs were incubated at 32°C, 37°C, or 39.5°C for 24 h and cell lysates were immunoblotted for PKCα. Representative of four similar blots and quantified band densities for PKCα normalized to β-tubulin and expressed as percent of levels in 37°C cells. (*B*) HEK 293T cells were transfected either with a combination of miRNA inhibitors against hsa-miR-27b-5p, hsa-miR-1260a, and hsa-miR-92a-1-5p or an equal concentration of a non-targeting scrambled-sequence inhibitor control. Transfected and untransfected cells were incubated at 32°C, 37°C, or 39.5°C for 24 h and immunoblotted for PKCα. A representative immunoblot and quantified band densities from four similar blots normalized to ß-tubulin and expressed as percent of levels in untreated 37°C cells are shown. Mean ± SEM. (*) *P* < 0.05, (†) *P* < 0.04 versus untreated 37°C and 39.5°C cells and miRNA inhibitor-treated 32°C cells, (¶) *P* < 0.02 versus untreated 39.5°C cells; *n* = 4.

Potential gene targets of the temperature-responsive miRNAs were identified using the miRTar algorithm (Hsu et al. 2011). Three of the five miRNAs, hsa-miR-92a-1-5p, hsa-miR-27b-5p, and hsa-miR-1260a, were predicted to target the PRKCA gene encoding PKCα, an important signaling molecule. To determine whether the observed temperature-responsive changes in miRNA expression are biologically relevant and impact expression levels of target protein, we analyzed the effects of hypo- and hyperthermia on PKCα protein levels in hSAECs (Fig. 4A) and HEK 293T cells (Fig. 4B) by Western blotting. Compared with 37°C HEK 293T cell cultures, levels of PKCα were 37% lower in cells incubated at 32°C for 24 h and 35% higher in cells incubated at 39.5°C. Human SAECs exhibited a similar pattern of temperature-dependent PKCα protein expression as the HEK 293T cells. To determine the potential contribution of the hypothermia-induced miRNAs to changes in PKCα protein levels, we pre-loaded HEK 293T cells with miRNA inhibitors against hsa-miR-92a-1-5p, hsa-miR-27b-5p, and hsa-miR-1260a for 24 h prior to a 24 h-exposure to 32°C, 37°C, or 39.5°C followed by Western blot analysis of PKCα protein levels (Fig. 4B). Cells that were preloaded with these three miRNA inhibitors increased PKCα levels at all three temperatures with the greatest increase occurring in the cells incubated at 32°C.

**FIGURE 5.**
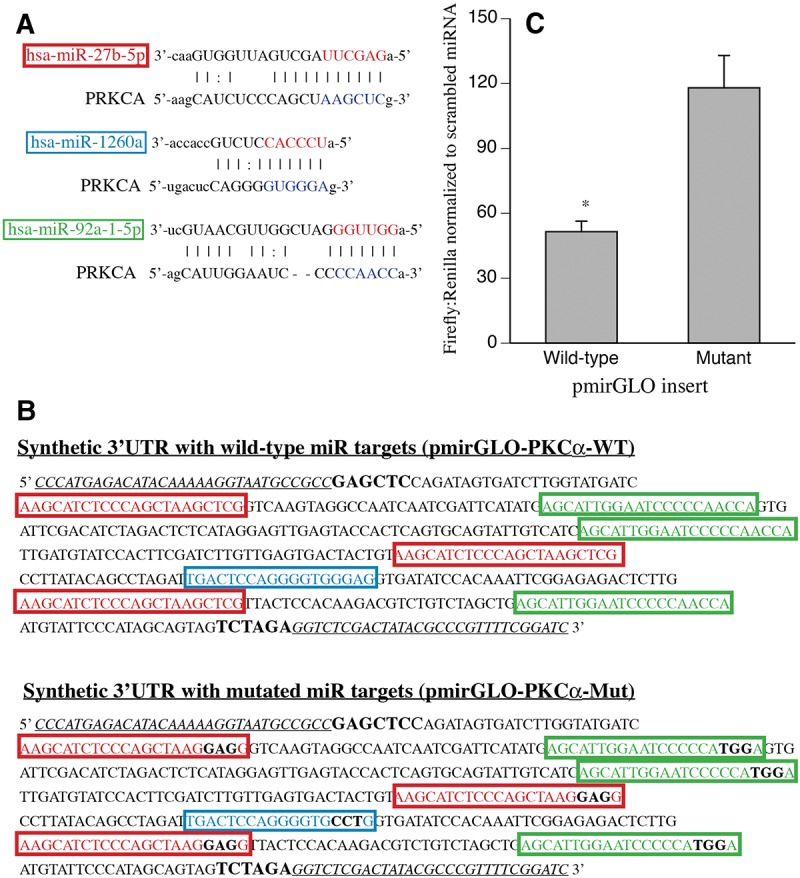
Functional analysis of the PKCα 3′ UTR binding sites for the temperature-sensitive miRNAs. (*A*) The putative binding sequences for hsa-miR-27b-5p, hsa-miR-1260a, and hsa-miR-92a-1-5p in PKCα 3′ UTR are shown. (*B*) Sequence of pmirGLO-PKCα-WT or pmirGLO-PKCα-Mut generated by cloning repeats of the putative miR binding sites or mutated sequences, respectively, into pmirGLO. The mutated nucleotides in each miR target sequence is indicated by black text for pmirGLO-PKCα-Mut. (*C*) HEK 293T cells were transfected with a combination of synthetic miRNA mimics for hsa-miR-27b-5p, hsa-miR-1260a, and hsa-miR-92a-1-5p or an equal concentration of non-targeting scrambled control mimic and pmirGLO-PKCα-WT (wild-type) or pmirGLO-PKCα-Mut (mutant), incubated at 37°C for 24 h, and dual luciferase assays were performed. Mean ± SEM. (*) *P* < 0.05, *n* = 4.

To further demonstrate the specificity of hsa-miR-92a-1-5p, hsa-miR-27b-5p, and hsa-miR-1260a for the PKCα 3′ UTR, multiple potential binding sites for these three miRNAs were identified in the 3′ UTR of PRKCA gene using PITA (Kertesz et al. 2007) and incorporated into pmirGLO dual luciferase miRNA reporter plasmid (Fig. 5A,B). pmirGLO-PKCα-WT contained three binding sites each for hsa-miR-92a-1-5p and hsa-miR-27b-5p and one binding site for hsa-miR-1260a and pmirGLO-PKCα-Mut contained an identical sequence except inactivating mutations were introduced into each of the putative miRNA binding sequences. HEK 293T cells were transfected with miRNA mimics for hsa-miR-92a-1-5p, hsa-miR-27b-5p, and hsa-miR-1260a or with scrambled mimic and with pmirGLO-aPKCa-WT or pmirGLO-PKCα-Mut and were lysed and analyzed for luciferase activities 48 h later (Fig. 5C). The miRNA mimics reduced firefly luciferase levels in cells transfected with pmirGLO-PKCα-WT by 49.5% compared with scrambled mimic. In contrast, the miRNA mimics had no effect on luciferase levels in cells transfected with pmirGLO-PKCα-Mut.

### Downstream biological consequences for changes in temperature

**FIGURE 6.**
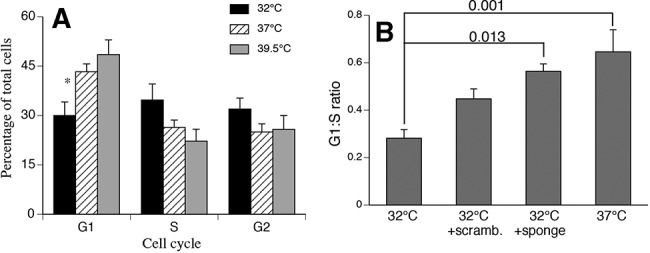
Effect of incubation temperature on cell-cycle progression. (*A*) Human SAECs were serum-starved for 24 h at 37°C, then incubated at 32°C, 37°C, or 39.5°C for an additional 24 h in serum-containing growth medium, stained with propidium iodide, and the proportion of cells in G1, S, and G2 phases was determined using flow cytometry. (*B*) HEK 293T cells were transfected with a combination of synthetic miRNA inhibitors for hsa-miR-27b-5p, hsa-miR-1260a, and hsa-miR-92a-1-5p or an equal concentration of non-targeting scrambled control inhibitor. After 24 h recovery, the cells were serum-starved for 24 h at 37°C, then incubated in serum-containing growth medium at 32°C or 37°C for 24 h, and cell-cycle analysis performed and the G1:S ratio calculated. Mean ± SEM, *n* = 5, (*) *P* < 0.05 in *A*. *P*-values are indicated in *B*.

To understand the potential biological impact of altered PKCα protein levels, we analyzed cell-cycle progression in growth-factor starved hSAECs incubated for 24 h at 32°C, 37°C, and 39.5°C in serum-containing growth medium (Fig. 6A). Human SAECs incubated at 32°C exhibited relatively fewer cells in G1 phase and more cells in S phase compared with 37°C and 39.5°C cells. To evaluate the contribution of the temperature-responsive PKCα-targeting miRNAs, we transfected HEK 293T cells with miRNA inhibitors against hsa-miR-92a-1-5p, hsa-miR-27b-5p, and hsa-miR-1260a 24 h prior to growth factor starvation at 32°C or 37°C, then replaced the medium with serum-containing growth medium for an additional 24 h (Fig. 6B). As we found for hSAECs, the G1:S ratio was lower in HEK 293T incubated at 32°C cells than in 37°C cells. Importantly, the temperature-dependent difference was reduced by pretreating 32°C cells with miRNA inhibitors.

## DISCUSSION

Deviations from the normal core temperature range may have important consequences for health and disease (Hasday 1996; Hasday et al. 2011; Shah and Hasday 2012; Singh and Hasday 2013). Although hypothermia is generally considered to be anti-inflammatory and pro-survival (Beilin et al. 1998; The Hypothermia after Cardiac Arrest Study Group 2002; Ning et al. 2002; Shankaran et al. 2005), moderate hypothermia can prolong TNFα and IL-1β expression (Fairchild et al. 2004, 2005), and increase expression of CD14 (Sonna et al. 2006) in mononuclear phagocytes. While moderate hyperthermia is usually considered pro-inflammatory (Ostberg et al. 2000, 2001; Hasday et al. 2003; Rice et al. 2005; Lipke et al. 2010, 2011), moderate hyperthermia reduces expression of TNFα (Singh et al. 2002; Cooper et al. 2010a,b), G-CSF (Zhang 2011), IL-1β (Cahill et al. 1996, 1997; Xie and Calderwood 2001; Xie et al. 2002), c-fos (Xie et al. 2003), and IL-18 (Wang et al. 2010). Our previous studies have demonstrated that modest deviations from normothermia can alter stress-induced and proinflammatory signaling pathways, including heat shock factor-1 (HSF-1), p38 MAP kinase, and NFκB (Fairchild et al. 2004, 2005; Tulapurkar et al. 2009, 2012; Shah et al. 2012) and modify expression of important genes. We have previously identified several mechanisms by which shifts in temperature within the physiologic range modify gene transcription (Singh et al. 2002, 2008; Cooper et al. 2010a, b). In this paper, we have expanded these studies by analyzing how shifts in temperature within the clinical range modify expression of a discrete subset of miRNAs and evaluating the consequences for post-transcriptional regulation, the effects on protein expression of an important predicted miRNA target, PKCα, and the impact on cell proliferation.

As the primary cultured hSAECs used in this study are isolated from airways that are ≤1 mm in diameter and close to core temperature in vivo (McFadden and Pichurko 1985), the in vitro exposure temperatures used in this study are similar to the temperatures these cells experience in vivo during clinically relevant hypothermic, normothermic, and hyperthermic states. The following four comparison groups were studied to simulate clinically relevant conditions: hypothermia (32°C versus 37°C), hyperthermia (39.5°C versus 37°C), therapeutic hypothermia (32°C with TNFα versus 37°C with TNFα), and fever (39.5°C with TNFα versus 37°C with TNFα). Microarray and Nanostring assays with confirmation by qRT-PCR identified only 5 (1%) of 505 miRNAs detected in hSAECs that exhibited temperature-dependent expression and this pattern was not influenced by the presence of TNFα.

Billeter et al. (2012) analyzed miRNA expression in ex vivo whole human blood stimulated with low dose (1 ng/mL) LPS using microarray and found only 3% of screened miRNAs showed temp-responsiveness to temperature changes between 34°C and 40°C. One of these was hsa-miR181a, which demonstrated a similar pattern to its expression in hSAECs, was increased in hypothermic cells and reduced in hyperthermic cells. Truettner et al. (2011) analyzed the effect of 4 h exposure to hypothermia or hyperthermia on miRNA expression in brain in a rat traumatic brain injury model. They found that post-trauma brain levels of miR-27b were higher in rats exposed to hypothermia than in normothermic rats. While these two studies showed similar effects of temperature shifts on expression of miR-181a and miR-27b as found in our current study, these authors did not consider whether the temperature-responsive miRNAs were the 3p- or 5p-arm. Nonetheless, these two studies and the results of our current study demonstrate that clinically relevant hypothermic temperatures induce expression of surprisingly few miRNA genes.

In our current study, miRNAs were profiled using two assay platforms that had significant overlap in the miRNAs probed but with important differences. While the Affymetrix microarray contains probe sets for both precursor and mature miRNA, the nCounter solution hybridization assay only contains probe sets for mature miRNA. The microarray includes probe sets for both strands of most mature miRNAs, while the nCounter probe sets are biased toward the more abundant (guide) strand of mature miRNA and probe for few star (less abundant “passenger”) strands. As most of the temperature-sensitive miRNAs detected by microarray were the star strands, these miRNAs would be largely undetectable by the nCounter probe set. None of the miRNAs that were detected by both platforms exhibited temperature-sensitive expression patterns. However, the temperature-responsive expression patterns detected for five miRNAs by either the microarray or nCounter method were confirmed by strand-specific qRT-PCR. Of these, hsa-miR-27b and hsa-miR-1260a exhibited much higher absolute expression levels, 18 and seven copies per cell, than the other three temperature-responsive miRNAs.

The mechanism by which expression levels of hsa-miR-92a-5p, hsa-miR-27a-5p, hsa-miR-27b-5p, hsa-miR-1260a, and hsa-miR-181a-3p increase in hypothermic cells is not clear. It is also not certain whether the same mechanism is responsible for the increase of all five miRNAs. MicroRNA precursors are transcribed by the same machinery that transcribes mRNA (Wang et al. 2010). Some such as hsa-miR-92a are expressed as a miRNA cluster from a polycistronic gene coding for multiple miRNAs (Olive et al. 2010). The sequence for hsa-miR-1260a precursor is embedded in exon 3 of the neuroglobin gene. Mature miRNA is processed stepwise from pri-miRNAs to ∼70 nt pre-miRNAs, ∼21 nt miRNA duplexes with protruding 2 nt 3′ ends, and finally to the single-strand mature miRNA, which is incorporated into the RISC (for review, see Filipowicz et al. 2008). Several lines of evidence from our study suggest that the expression profiles of most of these temperature-responsive mature miRNAs result from altered post-transcriptional processing. Quantitative RT-PCR analysis of the opposite strands and the pre- and pri-forms for three of the temperature-sensitive mature miRNAs, hsa-miR-92a-1, hsa-miR-27a, and hsa-miR-27b showed similar expression levels at all three temperatures. The Affymetrix microarray platform showed similar levels of 1260a pre-miRNA at all three temperatures (Table 1). In contrast, qRT-PCR analysis of hsa-miR-181a showed that both mature miRNA strands and the pre- and pri-forms showed a similar pattern of increased expression levels at 32°C and 39.5°C compared with 37°C, suggesting that the temperature-dependent expression of miR-hsa-181a-3p reflected altered transcription rates.

Quantitative RT-PCR demonstrated different temperature-dependent expression profiles for the star and guide strands for three of the temperature-responsive miRNAs (Fig. 3). While the two strands of Drosophila miRNA duplexes are loaded by different Argonaute proteins (Okamura et al. 2009; Ghildiyal et al. 2010), a similar Argonaute protein-specific miRNA strand sorting mechanism has not been demonstrated in mammalian cells. Considering past studies showing that the fate of each of the two strands in the miRNA duplex is influenced by its thermodynamic stability (Khvorova et al. 2003) and its interaction with Ago2 and Dicer when complexed with *trans*-activation response RNA-binding protein (TRBP) or protein activator of PKR (PACT) (Noland and Doudna 2013), strand selection is an attractive candidate for a temperature-sensitive step in miRNA processing. We found that the mature duplex forms of temperature-responsive miRNAs tended to have higher predicted stability and more asymmetry in stability than other miRNAs in their respective clusters and the temperature-responsive strands represented the usually less abundant strand (Noland and Doudna 2013). However, the mechanisms for temperature-dependent strand selection, the reason for its restriction to so few miRNAs, and the extensive bias toward star strands remains incompletely understood.

The potential consequence of temperature-dependent miRNA expression for target gene expression in hypothermic and hyperthermic conditions was investigated by analyzing protein expression levels of PKCα, which contains multiple predicted targets sequences for three of temperature-dependent miRNAs, hsa-miR-92a1-5p, hsa-miR-27b-5p, and hsa-miR-1260a, in its 3′ UTR region. We validated miRNA-binding activity of the putative miRNA-binding sites using dual luciferase vectors containing wild-type or mutated binding sites from PKCα. As animal miRNA may cause translational repression with or without mRNA decay (Brodersen and Voinnet 2009), we measured PKCα protein levels in hSAECs and HEK 293T cells exposed for 24 h to 32°, 37°C, and 39.5°C. As expected based on the increased expression of PKCα-targeting miRNAs, PKCα protein levels were significantly decreased in both the 32°C hSAECs and 32°C HEK293T cells compared with 37°C cell cultures. Furthermore, the effect of hypothermia on PKCα protein levels in HEK 293T cells was blunted by inhibitors against the three putative PKCα-targeting miRNAs. PKCα expression is known to be extensively regulated by miRNAs. The miRTar algorithm identified 372 miRNAs predicted to target the PRKCA 3′ UTR, including three of our temperature-responsive miRNAs. PRKCA targeting by hsa-miR-200b (Wang et al. 2014), hsa-miR-216b (Deng et al. 2013), hsa-miR-203 (Wang et al. 2013), and hsa-miR-24-2* (Martin et al. 2014) have been confirmed experimentally. PKCα levels have been shown to increase with increasing levels of hsa-miR-328 in A549 cells (Arora et al. 2011). PKCα has been reported to up-regulate hsa-miR-1 (Minetti et al. 2014), hsa-miR-101 (Chiang et al. 2010), and hsa-miR-15a (von Brandenstein et al. 2011). Our results show that three of the miRNAs that target PKCα are expressed at higher levels during hypothermia, which may contribute to unanticipated consequences of hypothermia exposure.

Considering that PKCα blocks G1/S and promotes G2/M transitions (Frey et al. 1992; Detjen et al. 2000; Gao et al. 2009; Poli et al. 2014), we compared cell-cycle profiles in hSAEC cells incubated for 24 h at 32°C, 37°C, and 39.5°C. Incubating hSAECs at 32°C, which reduces PKCα protein levels, reduced the proportion of cells in G1 and increased the proportion in S phase compared with 37°C cells. These results are consistent with a loss of G1/S transition block that would be expected with lower PKCα levels in the 32°C cells. Treatment with the same miRNA inhibitors that blunted the effect of hypothermia on PKCα protein levels also mitigated the effect of hypothermia on cell-cycle progression in HEK 293T cells. Although we cannot exclude other effects of hypothermia that modify cell cycling, these data suggest that the miRNA targeting of PKCα contributes to altered cell-cycle progression of cells exposed to clinically relevant hypothermia.

In summary, we have shown that clinically relevant temperature deviations can modify expression levels of a narrow subset of miRNAs, which appear to largely reflect altered post-transcriptional processing. Three of the miRNAs that increase in hypothermic cells target PKCα with a predicted impact on cell-cycle transition. These previously unappreciated effects of temperature shifts may have important consequences in clinical hypothermic and hyperthermic states.

## MATERIALS AND METHODS

### Cell culture and treatment

Primary cultured hSAECs were purchased from the ATCC and maintained in airway epithelial cell basal medium supplemented with the small airway cell growth kit (ATCC). To analyze miRNA expression, hSAECs (lot no. 58704924) isolated from a 37-yr-old Caucasian male were growth factor-starved by culturing in basal medium without growth factors for 24 h at 37°C and 5% CO_2_, then incubated in basal medium with or without 1 ng/mL rhTNFα (R&D Systems) at 32°C, 37°C, or 39.5°C for 24 h in automatic CO_2_ incubators. The incubators were certified to have <0.2°C temperature variation (Forma) and were calibrated for each experiment using an electronic thermometer (FLUKE Instruments model 5211). A total of five independent experiments were performed for the microarray and Nanostring nCounter assays (NanoString Technologies). Human SAECs from the original donor and a second lot of hSAECs from a 20-yr-old Caucasian female donor (lot no. 60747453) were similarly treated for confirmation of specific miRNA expression by quantitative RT-PCR. HEK 293T cells were obtained from ATCC and were maintained at 37°C in Dulbecco's Minimal Essential Medium (DMEM) with 4.5 g/L glucose and supplemented with 5% fetal bovine serum (FBS).

### RNA extraction and RNA quality control

Total RNA was extracted from treated cells using TRIzol (Life Technologies) as per the manufacturer's instructions. The purity and concentration of RNA samples were determined by absorbance at 260 and 280 nm measured using a dual beam UV spectrophotometer (Nanodrop; Thermo-Fisher Scientific) and RNA integrity was evaluated by capillary electrophoresis using the RNA 6000 Nano Lab-on-a-Chip kit and the Bioanalyzer 2100 (Agilent Technologies) as per the manufacturer's instructions. Only sets of samples with acceptable RNA quality for all six treatments were used for the miRNA analysis. MiRNA expression profiles in three of the five sets of RNA samples were analyzed by microarray hybridization. The same three sets of samples and one additional set of samples were analyzed using the Nanostring nCounter solution hybridization assay.

### Array hybridization and analyses

MiRNA microarray analysis was performed by Genome Explorations. RNA was processed and labeled according to standard RT-IVT methods. Labeled cRNA (15.0 µg) was fragmented by ion-mediated hydrolysis and hybridized for 16 h at 45°C to Affymetrix miRNA v2.0 array. The arrays were read and the log_2_ mean intensities were used for further analysis. Data were first filtered for human miRNA and miRNA probe sets with at least one detection call among the 18 samples were included for Perform Principal Components Analysis (PCA) to identify outlier samples. One 39.5°C sample was identified as an extreme outlier and removed from further analysis. ANOVA was performed for all six groups (with and without Benjamini-Hochberg FDR correction). Independent *t*-tests were performed for each pairwise comparison. Data were filtered for ANOVA *P* values <0.05 and an absolute log_2_ fold-change >0.585 (1.5-fold) and *t*-test *P* value <0.05 in each pairwise comparison were selected for further validation.

### Nanostring nCounter assays

A novel multiplex assay for miRNA expression was performed using nCounter miRNA Expression Assay Kits at NanoString Technologies. This method enables multiplexed direct digital counting of miRNA molecules (Geiss et al. 2008). To prepare miRNA molecules for hybridization in the nCounter assay, proprietary DNA sequences called miRtags were ligated to the mature miRNAs using bridging oligonucleotides. After ligation and purification, the tagged mature miRNAs were hybridized to color-coded reporter probes and biotinylated capture probes, immobilized onto a cartridge, and each immobilized miRNA was identified based on its color code and quantified. A total of 811 human and human-associated viral miRNAs were simultaneously assayed. All assays were performed using 100 ng of total RNA following the standard nCounter miRNA Assay Protocol. Hybridizations were performed automatically by mixing 5 µL of each miRNA multiplex assay with 20 µL NanoString nCounter reporter probe mix and 5 µL capture probe mix (30 µL total reaction volume), and hybridizing at 65°C for 18 h. The raw miRNA counts were analyzed using the nSolver software and normalized using the top 100 normalization method. The normalized values were then subject to two-way ANOVA and individual pair wise *t*-tests. miRNA with ANOVA *P* values <0.05 and fold-change >1.5-fold and *t*-test *P* value <0.05 in each pairwise comparison were selected for further validation. The capture efficiency is ∼1%.

### Real-time quantitative RT-PCR for miRNA

Complementary DNA was prepared from total RNA and real-time quantitative PCR was performed using Taqman miRNA assays for hsa-miR-92a-1-5p, hsa-miR-27b-5p, hsa-miR-18a, and hsa-miR-1260a as per manufacturer's instructions. qRT-PCR for hsa-miR-1260a and both mature strands of hsa-miR-181a, hsa-miR-27a, hsa-miR-27b, and hsa-miR-92a, and were analyzed using (Quanta Bioscience) qscript miRNA assays. The pre-forms of hsa-miR-181a, hsa-miR-27a, hsa-miR-27b, and hsa-miR-92a were analyzed using qPCR of the cDNAs prepared for each miRNA using the Quanta kit and primers designed to target pre-sequence outside the mature miRNA sequences. *Ct* values were normalized to hsa-miR-23a, which was shown to be unaltered under these treatment conditions by the nCounter assay, and fold-change versus 37°C was calculated using the delta–delta method (Livak and Schmittgen 2001). To quantify the primary transcripts for these miRNAs RNA from the same cell lysates were reverse transcribed using commercial reverse transcriptase and oligo(dT) primers (Promega) and primers designed to exclude pre-form sequence. The Ct values for the primary transcripts were normalized to GAPDH. The products of the Quanta qRT-PCR assays of the temperature-responsive mature miRNAs were isolated using Qiagen minElute columns, then cloned into the T-vector cloning plasmid (Promega) according to the manufacturer's protocol. Plasmids were prepared using Qiagen miniprep columns and DNA was sequenced using T7 primer (Eurofins Genomics).

### Estimation of absolute miRNA expression levels

The total number of mature hsa-miR-1260a and the opposite strands of the other temperature-responsive miRNAs, hsa-miR-27a-3p, hsa-miR-27b-3p, hsa-miR-92a-1-3p, and hsa-miR-181a-5p, were calculated from the Nanostring data using total number of hybridization events, assuming a collection efficiency of 1% (provided by Nanostring) and a total RNA isolation yield of 20 µg per million hSAECs. This method estimated let-7a-5p expression as 204 copies per hSAEC cell, which is similar to the reported let-7a-5p expression level in A549 cells of 200 copies per cell (Bakre et al. 2012).

### Thermodynamic analysis of temperature-responsive miRNAs

Thermodynamic stabilities of miRNA duplex and each of its ends comprising the terminal 3 bp and 2 nt 3′ overhangs were calculated using nearest-neighbor methods (Mathews et al. 1999) with adjustment for the composition of the 3′ overhang (O'Toole et al. 2005) using University of Vienna RNAFold Program (Gruber et al. 2008). For each miRNA, the total free energy of the duplex and the free energy with either the first 5 nt of the 5p arm or 3p arm forced to be unpaired was calculated. The free energy of each end was estimated by subtracting the free energy values of each terminal unpaired constraint from the total free energy. The absolute value of the difference in free energy of the two ends was calculated as a measure of thermodynamic asymmetry of the miRNA duplex.

### Transfection of miRNA inhibitors

HEK 293T cells were seeded in 6 × 35 mm cell culture dishes (Corning Biosciences) and transfected 24 h later with pEZX-AM03 vector containing complementary sequence against hsa-miR-92a1-5p, hsa-miR-27b-5p, and hsa-miR-1260a or scrambled sequence. Cells were incubated at 37°C for 24 h followed by incubation at 32°C, 37°C, or 39.5°C for next 24 h. Cells were lysed in RIPA at the end of 24 h and immunoblotting was performed as described below. A total of four independent experiments were performed.

### Interaction of temperature-sensitive miRNAs with PKCα 3′ UTR

A 406 nt DNA designed to include the predicted targets of temperature-sensitive miRNAs in PKCα 3′ UTR was synthesized as gBlocks and cloned between XbaI and SacI sites in pMiRGLO (Promega) to generate pmirGLO-PKCα-WT. This sequence contains three predicted binding sites each for hsa-miR-27b-5p and hsa-miR-92a-1-5p and one binding site for hsa-miR-1260a. A control plasmid, pmirGLO-PKCα-Mut, was similarly generated in which each of the predicted miRNA targeted sequences was mutated (Fig. 5B). HEK 293T cells were transfected with a combination of synthetic miR mimics for hsa-miR-92a1-5p (50 nM), hsa-miR-27b-5p (50 nM), and hsa-miR-1260a (25 nM) or scrambled sequence (125 nM) (Sigma MISSION microRNA Mimics) and 5 ng pmirGLO-PKCα-WT or pmirGLO-PKCα-Mut using Lipofectamine 2000 (Life Technologies). Cells were then incubated at 37°C for 48 h and lysed in Passive lysis buffer (Promega) and analyzed using a commercial dual luciferase assay (Promega) according to the manufacturer's protocol using a PerkinElmer Victor3 Multilabel Plate reader. The firefly to renilla luminescence ratio was calculated for each well and a mean luminescence ratio was calculated for each treatment group. A set of four independent experiments were performed.

### Analysis of PKCα protein expression

Human SAECs and HEK 293T cells were incubated at 32°C, 37°C, or 39.5°C for 24 h, lysed in RIPA buffer containing protease and phosphatase inhibitors, resolved by SDS-PAGE, transferred to PVDF membrane (Immobilon-FL Transfer Membrane, Millipore), blocked with Odyssey Blocking Buffer (LI-COR), probed with primary antibodies against protein kinase C (PKC)-α (Cell Signaling Tech. Cat#2056S) or β-tubulin (Millipore Cat#MAB3408) and IRDye 800CW Goat anti-Rabbit IgG, IRDye 680RD Goat anti-Mouse IgG (LI-COR) secondary antibodies. In some experiments, HEK 293T cells were transfected with inhibitors of hsa-miR-92a-1-5p, hsa-miR-27b-5p, and hsa-miR-1260a or with controls targeted to a scrambled sequence 24 h prior to changing culture temperature. The bands were detected and quantified using Odyssey-CLX infrared imaging system. The density of each PKCα band was expressed as a ratio to the β-tubulin band density from the same sample and was compared with 37°C control for each experiment.

### Cell-cycle analysis by flow cytometry

Human SAECs were cultured at 37°C in complete growth medium (ATCC) for 24 h, then were growth factor-starved by culturing in basal medium (ATCC) without growth factors for next 24 h at 37°C. The serum-starved cells were then either incubated in serum-free basal medium or complete growth medium containing 5% serum for an additional 24 h at 32°C, 37°C, or 39.5°C to analyze the effects of incubation temperature on cell cycle in the presence and absence of exogenous mitogen. Cells were harvested using trypsin-EDTA, fixed in cold 70% ethanol for 1 h at −20°C, washed twice with PBS and treated with RNAse (Qiagen). The cells were then stained with Propidium Iodide at a final concentration of 200 ng/mL (Sigma) and analyzed for cell cycle using a Becton-Dickinson Flow cytometer and the Watson cell-cycle algorithm in the manufacturer's FlowJo software. HEK 293T cells were transfected with miRNA inhibitors and subjected to the same cells-cycle analysis as hSAECs.

### Statistics

For quantitative RT-PCR and immunoblot data, differences between treatment groups were analyzed by repeat Mann–Whitney test using JMP 9 (SAS Institute, Inc). Differences among >2 treatment conditions was analyzed by one-way ANOVA. In some cases we applied a post hoc Tukey's Honestly Significant Difference test. Effects of temperature and TNFα were distinguished using a two-way ANOVA.
